# Synthesis of light needles with tunable length and nearly constant irradiance

**DOI:** 10.1038/s41598-018-21007-7

**Published:** 2018-02-08

**Authors:** Rosario Martínez-Herrero, David Maluenda, Ignasi Juvells, Artur Carnicer

**Affiliations:** 1 Departamento de Óptica, Universidad Complutense de Madrid, Facultad de Ciencias Físicas, Ciudad Universitaria, 28040 Madrid, Spain; 2 Departament de Física Aplicada, Universitat de Barcelona (UB), Facultat de Física, Martí i Franquès 1, 08028 Barcelona, Spain

## Abstract

We introduce a new method for producing optical needles with tunable length and almost constant irradiance based on the evaluation of the on-axis power content of the light distribution at the focal area. According to theoretical considerations, we propose an adaptive modulating continuous function that presents a large derivative and a zero value jump at the entrance pupil of the focusing system. This distribution is displayed on liquid crystal devices using holographic techniques. In this way, a polarized input beam is shaped and subsequently focused using a high numerical aperture (NA) objective lens. As a result, needles with variable length and nearly constant irradiance are produced using conventional optics components. This procedure is experimentally demonstrated obtaining a 53*λ*-long and 0.8*λ*-wide needle.

## Introduction

About sixty years ago, McLeod^1^ introduced conical lenses as a way to produce light axicons. Nowadays, optical needles are used in optical tweezers and in those techniques where long depth of focus is required. A variety of methods to achieve long needles with sub-wavelength width have been reported^2–16^. In general, most of this techniques are based on handling the discontinuities of the modulating distribution at the entrance pupil of the focusing system. The expected characteristics of an optical needle are small transverse width, negligible beam divergence and large longitudinal extension of the focal region. Note that polarization of the input electromagnetic field is also an important design variable. Very frequently, radial polarization is used because the produced beam displays the smallest spot size and a remarkable longitudinal polarization^17^.

The present paper aims to develop a procedure for generating needles with tunable axial extent and controlled on-axis uniformity. The incident beam is tailored by means of a special continuous modulating function designed to maximize the length of the needle according to on-axis irradiance considerations. This distribution is experimentally implemented by means of digital holography. Using the proposed technique, we produced in the laboratory a 53*λ*-long and 0.8*λ*-wide needle. Interestingly, these values are only limited by the characteristics of the electronic devices used in the optical setup. In other words, devices with improved features will be able to produce needles with better characteristics. Another remarkable characteristic of our design is that needles produced with linearly and radially polarized light have the same length.

This paper is organized as follows: first, after introducing key concepts in propagation of light at the the focal area, we propose a mathematical framework for producing long needles with almost constant irradiance. Then, a modulation function that fulfils the mathematical properties of our approach is suggested. This formalism is analysed by means of computer simulations. Later on, we describe the optical system required for producing such needles and some experimental results are obtained and discussed. Finally, we present our conclusions. Moreover, in the Methods section we provide mathematical details on the design properties of the modulation function.

## Results

### Needle design

**Figure 1:**
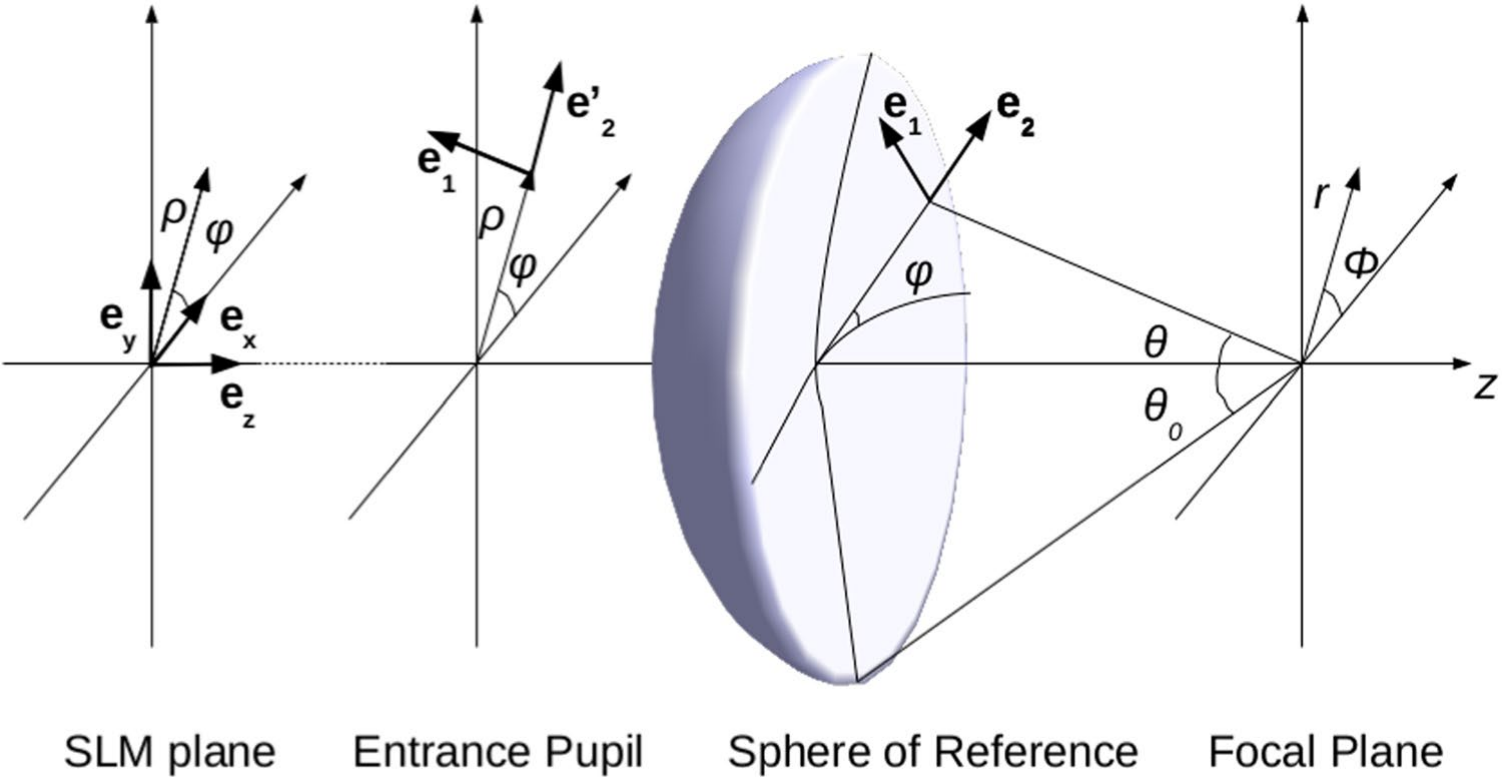
Coordinate systems and geometrical variables.

Let us first consider a monochromatic beam at the entrance pupil of an aplanatic high NA focusing system. The electric field **E** = (*E*_*x*_, *E*_*y*_, *E*_*z*_) at the focal area is described by the Richards-Wolf integral^18^

1 $${\bf{E}}(r,\varphi ,z)=A\,{\int }_{0}^{{\theta }_{0}}\,{\int }_{0}^{2\pi }\,{{\bf{E}}}_{0}(\theta ,\phi ){e}^{ikr\sin \theta \cos (\varphi -\phi )}{e}^{-ikz\cos \theta }\,\sin \,\theta \,{\rm{d}}\theta \,{\rm{d}}\phi $$

where *A* is a constant, *k* is the wave number, (*r*, *ϕ*, *z*) are the cylindrical coordinates at the focal area, *θ* and *φ* are the polar and the azimuthal angles at the Gaussian sphere of reference, *θ*_0_ = max{*θ*} is the semi-aperture angle and NA = sin *θ*_0_. **E**_0_ is the vector angular spectrum described by

2 $${{\bf{E}}}_{0}=\sqrt{\cos \,\theta }(({{\bf{E}}}_{s}\cdot {{\bf{e}}}_{1})\,{{\bf{e}}}_{1}+({{\bf{E}}}_{s}\cdot {{\bf{e}}{\boldsymbol{^{\prime} }}}_{2})\,{{\bf{e}}}_{2})$$

where **e**_1_ and **e**_2_ are unit orthogonal vectors on the azimuthal and radial directions

3a $${{\bf{e}}}_{1}=(-\sin \,\phi ,\,\cos \,\phi ,0)$$

3b $${{\bf{e}}}_{2}=(\,\cos \,\theta \,\cos \,\phi ,\,\cos \,\theta \,\sin \,\phi ,\,\sin \,\theta )\,,$$

and ${{\bf{e}}{\boldsymbol{^{\prime} }}}_{2}=(\cos \,\phi ,\,\sin \,\phi ,0)$ is the projection of **e**_2_ on the entrance pupil plane (see Fig. 1 for details). **E**_*s*_ describes the transversal beam distribution at the entrance pupil of the optical system. **E**_*s*_ is described as the product of the illuminating beam profile *g*(*θ*) (with polarization **p**(*φ*)) and a certain modulation function *h*(*θ*) that is used to tailor the beam according to the requirements of the problem. Therefore,

4 $${{\bf{E}}}_{s}(\theta ,\phi )=g(\theta )\,h(\theta )\,{\bf{p}}(\phi \mathrm{).}$$

In this paper *g*(*θ*) is considered Gaussian.

In order to design a light needle, the resulting field **E** on the *z*-axis is taken into account, i.e.:

5 $${\bf{E}}\mathrm{(0},z)={\int }_{0}^{{\theta }_{0}}\,\tilde{{\bf{F}}}(\theta ){e}^{-ikz\cos \theta }\,\sin \,\theta \,{\rm{d}}\theta $$

with

6 $$\tilde{{\bf{F}}}(\theta )=A\,{\int }_{0}^{2\pi }\,{{\bf{E}}}_{0}(\theta ,\phi )\,{\rm{d}}\phi =A\,{\int }_{0}^{2\pi }\,\sqrt{\cos \,\theta }(({{\bf{E}}}_{s}\cdot {{\bf{e}}}_{1})\,{{\bf{e}}}_{1}+({{\bf{E}}}_{s}\cdot {{\bf{e}}{\boldsymbol{^{\prime} }}}_{2})\,{{\bf{e}}}_{2})\,{\rm{d}}\phi $$

Our target is to determine the beam **E**_*s*_ at the entrance pupil of the objective lens that produces a light distribution at region Ω = (−*L*, *L*) around the focus *z* = 0 with |**E**(0, *z*)|^2^ taking significant values. For this purpose, we introduce the power-content ratio *q* defined as

7 $$q=\frac{{\int }_{-L}^{L}\,|{\bf{E}}\mathrm{(0},z{)|}^{2}\,{\rm{d}}z}{{\int }_{-\infty }^{\infty }\,|{\bf{E}}\mathrm{(0},z{)|}^{2}\,{\rm{d}}z}.$$

It can be demonstrated that the half-length of the needle *L* and the ratio *q* are related by means of the following inequality:

8 $$1-q\le \frac{2\lambda }{I{L}^{2}{k}^{2}}\,[{\int }_{{\alpha }_{0}}^{1}\,{|\frac{{\rm{d}}\tilde{{\bf{F}}}(\alpha )}{{\rm{d}}\alpha }|}^{2}\,{\rm{d}}\alpha +\frac{|\tilde{{\bf{F}}}({\alpha }_{0}{)|}^{2}}{1-{\alpha }_{0}}]+\frac{\mathrm{8|}\tilde{{\bf{F}}}({\alpha }_{0}{)|}^{2}}{IL{k}^{2}}$$

where *α* = cos *θ*, *α*_0_ = cos *θ*_0_, and $I={\int }_{-\infty }^{\infty }\,|{\bf{E}}\mathrm{(0},z{)|}^{2}\,{\rm{d}}z$; moreover, we assume that $\tilde{{\bf{F}}}\mathrm{(1)}=0$. This expression is derived in the Methods section and represents the main theoretical result of this paper. Interestingly, Eq. (8) provides an analytic procedure to determine the size of the region around the focus where a fraction *q* of the energy is concentrated.

Equation (8) depends on two terms: the derivative of $\tilde{{\bf{F}}}(\alpha )$ and the jump of function $\tilde{{\bf{F}}}$ at the entrance pupil of the objective lens, $\tilde{{\bf{F}}}({\alpha }_{0})$. In this work, $\tilde{{\bf{F}}}(\alpha )$ is a continuous function selected in such a way that fulfils two conditions: $\tilde{{\bf{F}}}(\alpha )$ presents a zero jump at the entrance pupil and (ii) $\frac{{\rm{d}}\tilde{{\bf{F}}}(\alpha )}{{\rm{d}}\alpha }$ displays very large values. Accordingly, Eq. (8) is simplified to give

9 $$1-q\le \frac{\lambda }{I{L}^{2}{k}^{2}}\,{\int }_{{\alpha }_{0}}^{1}\,{|\frac{{\rm{d}}\tilde{{\bf{F}}}(\alpha )}{{\rm{d}}\alpha }|}^{2}\,{\rm{d}}\alpha \mathrm{.}$$

For instance, if Ω encloses at least the 75% of the total on-axis power content (*q* = 0.75), *L* can be calculated from

10 $$L=\frac{\lambda }{\pi }\,{[\frac{{\int }_{{\alpha }_{0}}^{1}{|\frac{{\rm{d}}\tilde{{\bf{F}}}(\alpha )}{{\rm{d}}\alpha }|}^{2}{\rm{d}}\alpha }{{\int }_{{\alpha }_{0}}^{1}|\tilde{{\bf{F}}}(\alpha {)|}^{2}{\rm{d}}\alpha }]}^{1/2}.$$

Those functions *h*(*θ*) that produces distributions $\tilde{{\bf{F}}}(\alpha )$ that fulfils the previous requirements are candidates to be selected for producing long light distribution along the *z*-axis. Provided that *h*(*θ*) is known, $\tilde{{\bf{F}}}(\alpha )$ is determined using Eq. 6 and then, the needle length 2*L* can be theoretically estimated using Eq. (10). In this paper, we use the modulation function described by

11 $$h(\theta )=N\,{\rm{sinc}}\,(2\pi N\frac{\cos \,\theta -\alpha (m)}{1-{\alpha }_{0}})\,\sin \,\theta $$

where *α*(*m*) is

12 $$\alpha (m)=\frac{m}{2N}(1-{\alpha }_{0})+{\alpha }_{0},$$

sinc (*x*) = sin (*x*)/*x* is the unnormalized cardinal sine function, and *N* and *m* are natural numbers that fulfil 0 < *m* < 2*N*. In particular, large values of N produce long needles, *α*(*m*) is related to the transverse width and *h*(0) = *h*(*θ*_0_) = 0. It is worth to point out that Čižmár and Dholakia proposed a similar modulation function for generating Bessel beams in a paraxial scalar scenario^19^.

### Needle properties analysis

**Figure 2:**
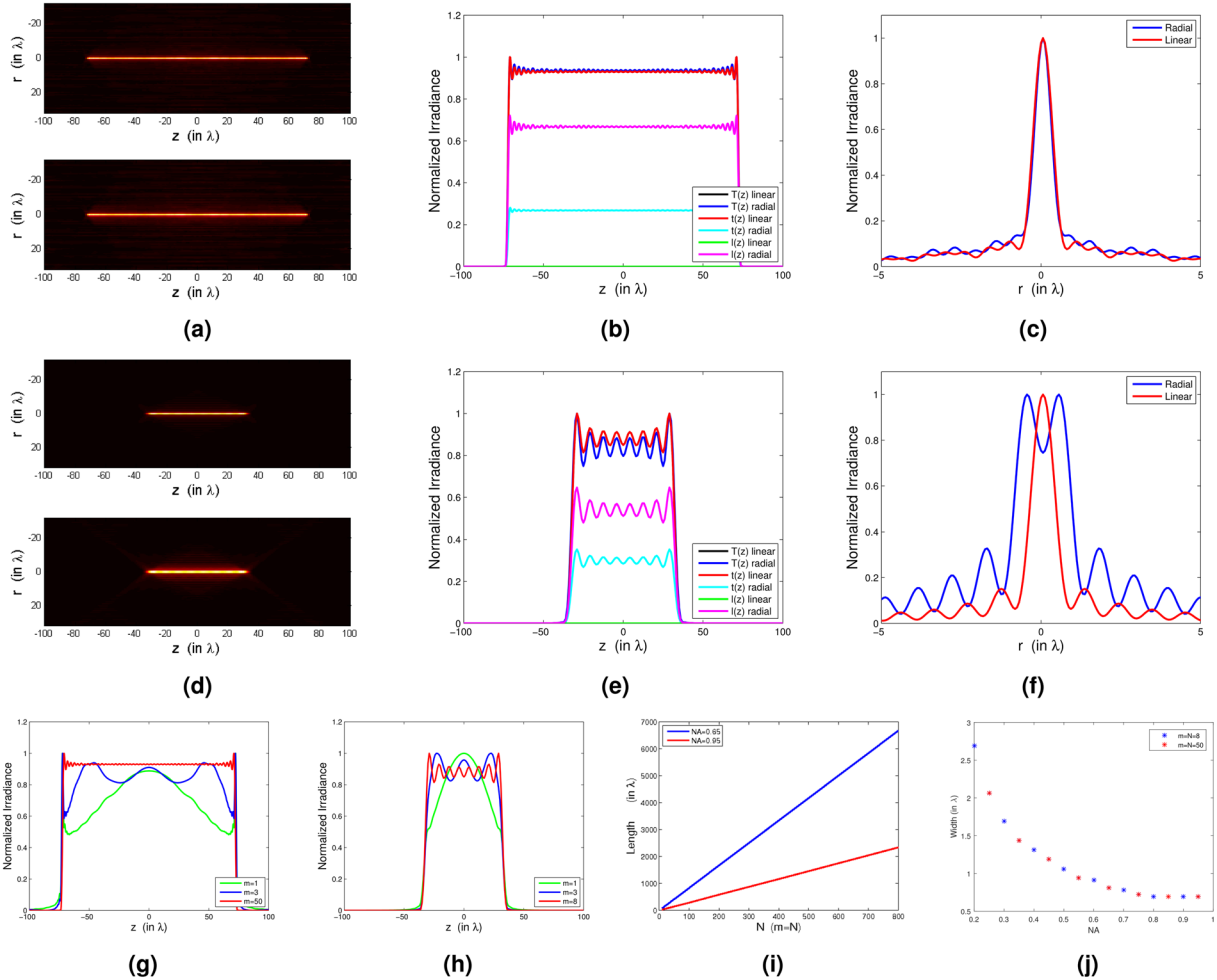
Numerical considerations on the needle design. Upper row: *N* = 50, *m* = 50, NA = 0.95. (**a**) False colour representation of *I*(*r*, *z*) using linear and radial polarization. (**b**) Distributions *T*(*z*), *t*(*z*) and *l*(*z*). (**c**) Transverse width *I*(*r*, *z* = 0). Central row *N* = 8, *m* = 8, NA = 0.65: (**d**) False colour representation of *I*(*r*, *z*) using linear and radial polarization. (**e**) Distributions *T*(*z*), *t*(*z*) and *l*(*z*). (**f**) Transverse width *I*(*r*, *z* = 0). Lower row: (**g**) *T*(*z*) for *N* = 50 and *m* = 1, 3, 50 (NA = 0.95). (**h**) *T*(*z*) for *N* = 8 and *m* = 1, 3, 8 (NA = 0.65). (**i**) Needle length as a function of *N* (*m* = *N* and NA = 0.65, 0.95). (**j**) Needle width as a function of NA (*m* = *N* and *N* = 8, 50).

To provide a better understanding on how the parameters of modulation function *h*(*θ*) (Eqs (11) and (12)) have to be selected, we have included several simulations using our design (see Fig. 2). For comparison purposes, we introduce the following parameters:

- transverse integrated irradiance *t*(*z*),
  13 $$t(z)=\int \,[{|{E}_{x}(r,\varphi ,z)|}^{2}+{|{E}_{y}(r,\varphi ,z)|}^{2}]\,r\,dr\,d\varphi ,$$
- longitudinal integrated irradiance *l*(*z*)
  14 $$l(z)=\int \,[{|{E}_{z}(r,\varphi ,z)|}^{2}]\,r\,dr\,d\varphi \,$$
- and total integrated irradiance *T*(*z*) = *t*(*z*) + *l*(*z*).

Figure 2(a) show a false colour representation of the needle angular-averaged irradiance *I*(*r*, *z*) for NA = 0.95, *N* = 50 and *m* = 50 for linear and radial polarization. Note that the length of the needle is almost independent of the polarization of the input illumination. Figure 2(b) show distributions *t*(*z*), *l*(*z*) and *T*(*z*) for the linear and radial cases with *N* = *m* = 50 and NA = 0.95. Interestingly, the behaviour of *T*(*z*) is very similar for the two polarizations considered and *l*(*z*) ≈ 0 for the linearly polarized case. Figure 2(c) displays the width of the needle *I*(*r*, *z* = 0).

Figure 2(d–f) show the angular-averaged irradiance *I*(*r*, *z*), distributions *t*(*z*), *l*(*z*) and *T*(*z*) and the width of the needle *I*(*r*, *z* = 0) for the linear and radial cases. These needles are calculated in the same conditions as in the experiment: *N* = 8, *m* = 8 and NA = 0.65. Note that for radial polarization, the maximum value of the needle is not achieved on the *z*–axis [Fig. 2(f)].

Figure 2(g–j) analyse the effect of different parameters on the behaviour of the needle for the linear polarized case. In Fig. 2(g,h) we illustrate the dependence of parameter *m* on the profile *T*(*z*) for *N* = 50, NA = 0.95 and *N* = 8, NA = 0.65, respectively. For *m* = 1 profiles are bell-shaped whereas the needle tends to be constant on the *z*–axis for *m* = *N*. Figure 2(i) displays the behaviour of length 2*L* as a function of *N*: it is apparent that the length of the needle is proportional to N. Finally, in Fig. 2(j) the needle width as a function of NA for *N* = 8, 50 and *m* = *N* is shown: sub-wavelength width is possible for high NA values but note that the width is independent of parameter *N*.

### Experimental setup

**Figure 3:**
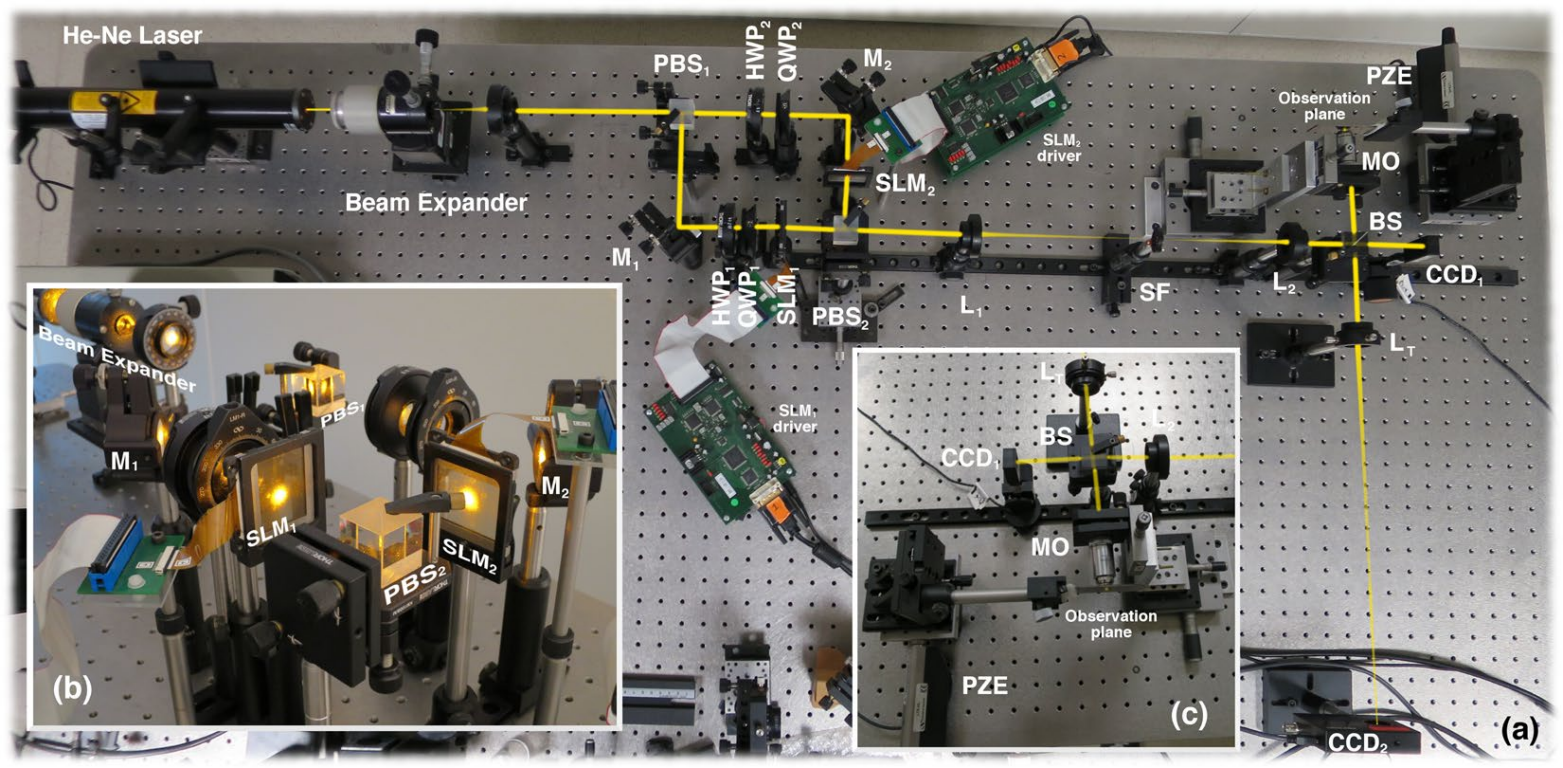
Experimental setup: PBS: polarizing beam splitter, M: mirror, HWP: half wave plate, QWP: quarter wave plate, SLM: spatial light modulator, L: lens, SF: spatial filter, BS: non-polarizing beam splitter, MO: microscope objective, PZE: piezoelectric stage for fine displacements, CCD: camera.

In order to provide an experimental verification of our theoretical approach, we produced needles using linear and radially polarized beams. These light distributions were created with the help of an optical system able to tailor amplitude and polarization of the input beam and a high numerical aperture objective lens. This set-up is based on a modified version of a Mach-Zehnder system: each transverse component of the input beam passes through one of the arms of the interferometer where computer generated holograms displayed on liquid crystal devices modulate each component of the input beam. Then, both components are recombined and subsequently focused using a microscope objective lens. An extended description of the optical arrangement can be found in^20,21^ whereas the holographic encoding method is described in^22^. The optical setup used is depicted in Fig. 3(a); insets 3(b,c) show the interferometric and recording parts of the system. The light source is a linearly polarized TEM_00_ *λ* = 594 nm He-Ne laser with the polarization direction set at 45° with respect to the *x*–axis. The beam is split using a polarized beam splitter (PBS_1_ on Fig. 3).

Each component of the beam **E**_*S*_ is independently modulated by holograms *h*_*x*_(*θ*,*φ*) and *h*_*y*_(*θ*,*φ*) displayed on two inexpensive Holoeye HEO 0017 spatial light modulators (SLM_1_ and SLM_2_). The wave plates in the optical system are used to rotate the oscillating plane and set-up the modulators to the required modulation curve. These displays have been calibrated using the method described in^23^; the corresponding modulation curves are shown in the Methods section. Holograms *h*_*x*_(*θ*,*φ*) and *h*_*y*_(*θ*,*φ*) are related with modulation function *h*(*θ*) by means of

15 $$({h}_{x}(\theta ,\phi ),{h}_{y}(\theta ,\phi ))=h(\theta )\,{\bf{p}}(\phi )$$

**Figure 4:**
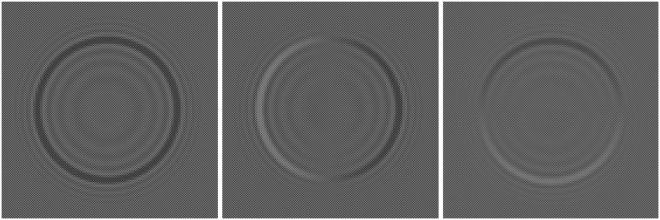
(Left) Computer generated hologram *h*_*x*_(*θ*, *φ*) displayed on SLM_1_ to encode the linear polarized needle (SLM_2_ remains unused in this case). (Center) Computer generated hologram *h*_*x*_(*θ*, *φ*) displayed on SLM_1_ to encode the *x*-component of the radially polarized needle. (Right) Hologram *h*_*y*_(*θ*, *φ*) displayed on SLM_2_ to encode the *y*-component of the radially polarized needle. Codification details can be appreciated if the figure is zoomed.

The polarization vector of the input beam **E**_*s*_ are **p** = (1, 0) and **p** = (cos *φ*, sin *φ*) for the linearly and radially polarized cases, respectively. Distributions *h*_*x*_(*θ*, *φ*) and *h*_*y*_(*θ*, *φ*) are encoded on the displays using the cell-based double-pixel hologram^22^. Figure 4 shows the encoded computer generated holograms used in this paper. Note that the holographic structure can be appreciated if the image is zoomed. As explained in the previous section, long needles are obtained for high values of *N*; if the condition *m* ≈ *N* holds, needles with a nearly constant profile are obtained. Moreover, the maximum feasible value for *N* is only limited by the resolution of the devices used to display the modulation function *h*(*θ*).

Then, the resulting distributions are subsequently recombined by means of polarized beam splitter 2 (PBS_2_). According to Eq. (4), **E**_*s*_ reads

16 $${{\bf{E}}}_{s}=\frac{1}{\sqrt{2}}\,\exp \,(-\frac{{\sin }^{2}\,\theta }{{f}_{0}^{2}\,{\sin }^{2}\,{\theta }_{0}})\,({h}_{x}(\theta ,\phi ){{\bf{e}}}_{x}+{h}_{y}(\theta ,\phi ){{\bf{e}}}_{y}),$$

where **e**_*x*_ = (1, 0, 0) and **e**_*y*_ = (0, 1, 0) (as indicated in Fig. 1); *f*_0_ = 1.5 is the filling factor (experimentally estimated). Finally, **E**_*s*_ is imaged and scaled on the entrance pupil of the objective lens using relay lenses L_1_ and L_2_.

The spatial filter removes non-desired off-axis diffracted terms. Note that the irradiance |**E**_*S*_|^2^ can be recorded by CCD_1_. This camera is useful to analyse the shape of |**E**_*S*_|^2^ or to determine the Stokes images at the entrance pupil of the microscope lens. In this case, an extra polariser should be located in front of the camera.

Finally, the beam is focused using a microscope objective with NA = 0.65. Camera CCD_2_ is used to image the focal area in combination with tube lens L_*T*_ with a focal length *f*_*T*_ = 400 *mm*. The position of the observation plane *z* is tuned by means of a Newport LTA-HL actuator with a minimum incremental motion *δ*_*z*_ = 50 *nm* and a repeatability *ε*_*z*_ = ±100 *nm*.

### Experimental results

**Figure 5:**
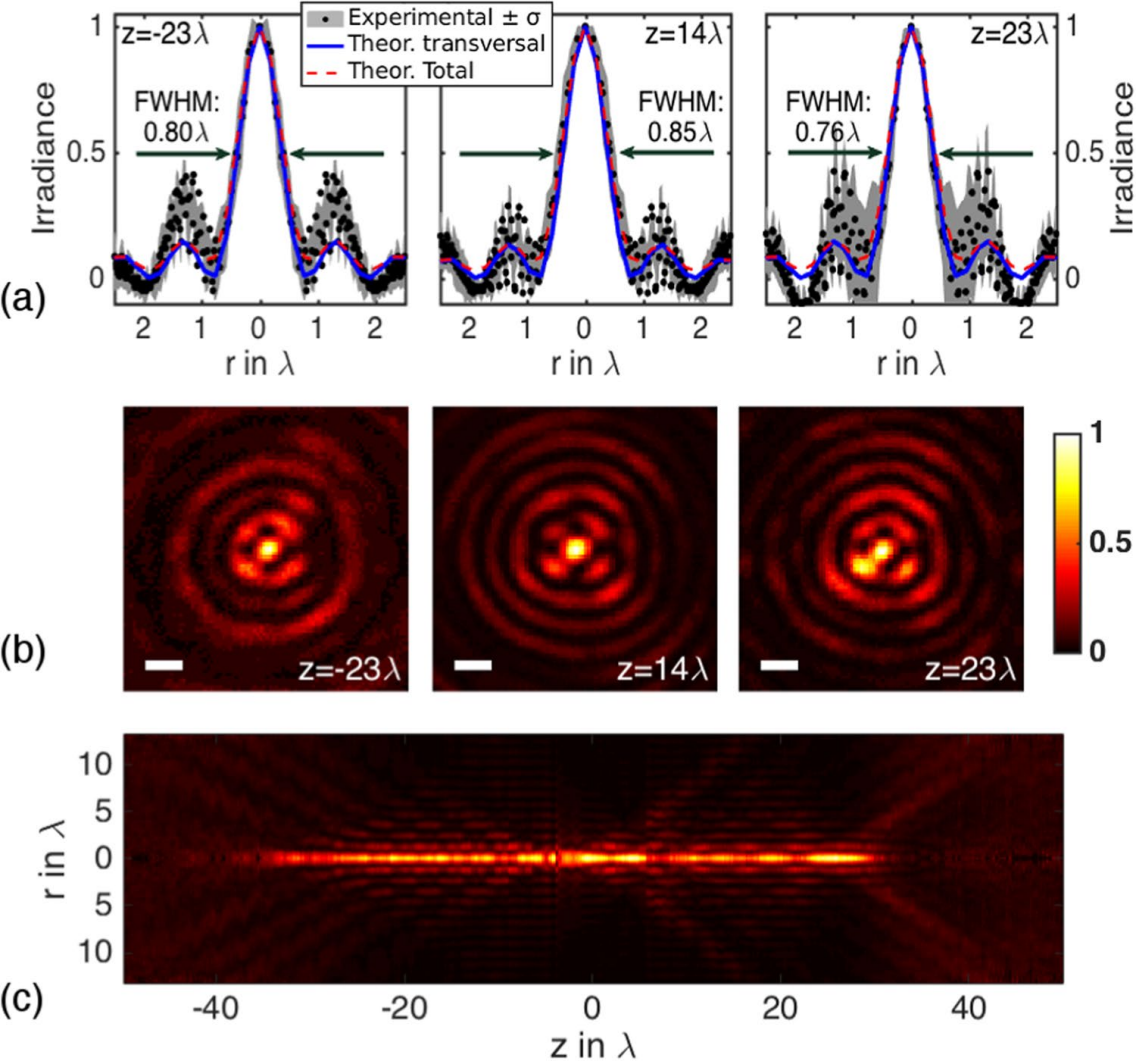
Experimental results with input linear polarization (*N* = 8, *m* = 8 and NA = 0.65): (**a**) Angular-averaged profiles *I*(*r*, *z*) and (**b**) irradiance of the imaged field |**E**(*r*, *ϕ*, *z*)|^2^ at *z* = −23*λ*, 14*λ*, 23*λ*; the white bar is equivalent to 1 *μm*. (**c**) Angular-averaged irradiance map *I*(*r*, *z*). Note that the needle length is ~53*λ*.

First, the synthesized beam at the entrance pupil **E**_*s*_ is linearly polarized. In such conditions the holograms for the *x*- and *y*- directions are *h*_*x*_(*θ*, *φ*) = *h*(*θ*) and *h*_*y*_(*θ*, *φ*) = 0. Parameters *N* and *m* are set to *N* = *m* = 8 and the focusing lens is NA = 0.65. Experimental irradiances |**E**(*r*, *ϕ*, *z*)|^2^ are recorded at planes *z* normal to the optical axis. These distributions are imaged on the CCD camera with the help of a long focal length lens. Figure 5(a) shows angular averaged profiles $I(r,z)\propto \int \,{|{\bf{E}}(r,\varphi ,z)|}^{2}\,d\varphi$ recorded at *z* = −23*λ*, 14*λ* and 23*λ*. Black dots and grey bands indicate the averaged values and the corresponding standard deviation. For comparative purposes, blue (transverse irradiance of the beam) and red (total irradiance) curves show numerical calculations carried out using Eq. 1. The width of the needle is obtained by calculating the Full Width at Half Maximum (FWHM) value of these curves. Accordingly, the estimated value is FWHM ~0.8*λ*. Figure 5(b) displays the corresponding recorded irradiances |**E**(*r*, *ϕ*, *z*)|^2^ at *z* = −23*λ*, 14*λ* and 23*λ*. In order to provide an account of the length of the needle, a series of irradiances |**E**(*r*, *ϕ*, *z*)|^2^ have been recorded for *z* ranging from −50*λ* to 50*λ* every Δ*z* = 148 *nm* ≈ *λ*/4. This information is used to produce a visual representation *I*(*r*, *z*) (see Fig. 5(c)). Interestingly, the estimation of the needle length using the FWHM is ~53*λ*, in agreement with the theoretical prediction stated in the ‘Needle properties analysis’ section. It should be pointed out that axial distortion can be seen in the needle. This undesirable behaviour is due to several combined effects. First, SLM modulation curves display calibration errors (see Methods: Measurement of the modulation response of the displays) that are propagated in the holographic encoding procedure. Second, set-up misalignments, lack of flatness of the optical components (polarisers, wave plates, mirrors, et cetera) or stage drifts also deteriorates the quality of the needle. All in all, spherical aberration is present in our system. Interestingly, this aberration severely deteriorates the needle profile as it has been reported in refs^11,24^.

**Figure 6:**
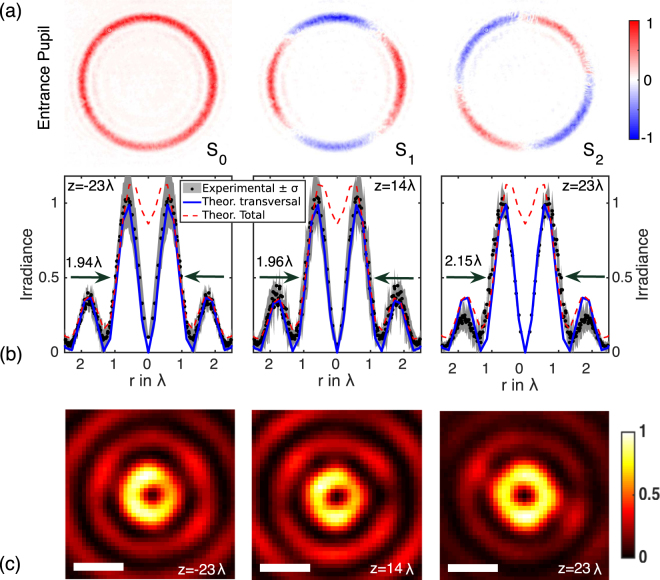
Experimental results with input radial polarization (*N* = 8, *m* = 8 and NA = 0.65): (**a**) Stokes images of the beam at the entrance pupil of the microscope objective. (**b**) Angular-averaged profiles *I*(*r*, *z*) and (**c**) irradiance of the imaged field |**E**(*r*, *ϕ*, *z*)|^2^ at *z* = −23*λ*, 14*λ*, 23*λ*; the white bar is equivalent to 1 *μm*.

Second, a longitudinally polarized needle has been produced. In this case, the input beam **E**_*S*_ is radially polarized and thus, the holograms are *h*_*x*_(*θ*, *ϕ*) = *h*(*θ*) cos *φ* and *h*_*y*_(*θ*, *ϕ*) = *h*(*θ*) sin *φ*. Again, *N* = *m* = 8. In order to test the polarization state of the input beam, the Stokes parameters *S*_0_, *S*_1_ and *S*_2_ has been measured at the entrance pupil of the focusing lens. The resulting Stokes images are shown in Fig. 6(a). Results clearly show that the beam **E**_*S*_ is radially polarized.

As in the previous case, the irradiance |**E**(*r*, *ϕ*, *z*)|^2^ has been recorded for *z* ranging from −50*λ* to 50*λ*. Nevertheless, longitudinally polarized needles cannot be visualized using conventional imaging optics. In our setup, the beam in the focal area is back-propagated trough the microscope objective and imaged on the CCD camera with the help of a long-focal length lens. In such conditions the longitudinal component is not propagated across the optical system and thus, it cannot be detected (see, for instance refs^25,26^). Despite the needle exists, a pipe rather than a needle is detected.

Angular-averaged profiles *I*(*r*, *z*) at *z* = −23*λ*, 14*λ*, 23*λ* are presented in Fig. 6(b). Again, blue and red-dashed curves show the theoretical predictions of the transverse and the total irradiance of the light needle calculated using the Richards-Wolf integral [Eq. (1)]; the black dots indicate experimental values. It is apparent that instead of a bell-shaped distribution a donought-like profile is recorded: in particular, the experimental values are fitted using the theoretical estimation of the transverse part of the beam. Note that the width of the beam remains constant, around 2*λ*. Finally, Fig. 6(c) shows the irradiance of the imaged field |**E**(*r*, *ϕ*, *z*)|^2^ at the above referred planes.

## Concluding Remarks

In summary, we proposed a new approach for producing high quality needles. First, we developed a theoretical framework for evaluating the length of an optical needle taking into account on-axis power-content constrains. Second, we introduced a specific modulating function that depends on several parameters that are used for tuning the length of the needle. The behaviour of the needles was analysed using computer calculations. Finally, experimental needles were optically implemented by means of digital holography techniques using polarized input beams. In particular, we produced a linearly polarized 53*λ*-long needle with sub-wavelength width. The proposed approach was also tested with a radially polarized input beam. Despite the resulting light distribution presents a very intense longitudinal component, the full needle cannot be recorded using conventional imaging optics.

## Methods

### Power content of an enclosed region

In order to assess the length of the region around the focus (*z* = 0) where the power-content ratio of |**E**(0, *z*)|^2^ is significant, it is necessary to derive a suitable criterion. This problem was extensively investigated some years ago using the so-called irradiance-moments framework^27^. The utility of this framework was confirmed because it is currently adopted as a ISO standard^28^. Nevertheless, this formalism is difficult to be implemented with fields such as the proposed one [Eq. (5)] because convergence of integrals is not guarantee^29^.

The target of this section is to derive an expression to evaluate *L* for a given *q*. Equation (5) can be simplified using the change of variable *α* = cos *θ*, i.e.:

17 $${\bf{E}}\mathrm{(0,}z)={\int }_{{\alpha }_{0}}^{1}\,\tilde{{\bf{F}}}(\alpha )\,{e}^{-ikz\alpha }\,{\rm{d}}\alpha $$

with *α*_0_ = cos *θ*_0_. It is important to recall that it is assumed that $\tilde{{\bf{F}}}\mathrm{(1)}=0$. For convenience, we introduce auxiliary vector **q**(*α*) defined as

18 $${\bf{q}}(\alpha )=\tilde{{\bf{F}}}({\alpha }_{0})\frac{1-\alpha }{1-{\alpha }_{0}}.$$

Note that $\tilde{{\bf{F}}}({\alpha }_{0})$ represents the jump discontinuity of function $\tilde{{\bf{F}}}(\alpha )$ at the entrance pupil. By using **q**(*α*), Eq. (17) can be rewritten as the combination of two terms **E**(0, *z*) = **E**_1_(0, *z*) + **E**_2_(0, *z*) namely

19a $${{\bf{E}}}_{1}\mathrm{(0},z)={\int }_{{\alpha }_{0}}^{1}\,[\tilde{{\bf{F}}}(\alpha )-{\bf{q}}(\alpha )]\,{e}^{-ikz\alpha }\,{\rm{d}}\alpha $$

19b $${{\bf{E}}}_{2}\mathrm{(0},z)={\int }_{{\alpha }_{0}}^{1}\,{\bf{q}}(\alpha )\,{e}^{-ikz\alpha }\,{\rm{d}}\alpha \mathrm{.}$$

The second order intensity-moment 〈*z*^2^〉_1_ for **E**_1_(0, *z*) is mathematically well defined and it can be calculated by means of the following expression:

20 $${\langle {z}^{2}\rangle }_{1}=\frac{{\int }_{{\alpha }_{0}}^{1}\,{|\frac{{\rm{d}}\tilde{{\bf{F}}}(\alpha )}{{\rm{d}}\alpha }|}^{2}\,{\rm{d}}\alpha }{{k}^{2}\,{\int }_{{\alpha }_{0}}^{1}\,{|\tilde{{\bf{F}}}(\alpha )|}^{2}\,{\rm{d}}\alpha }.$$

It is well known that the region around the focus ${\rm{\Omega }}=(-2\sqrt{{\langle {z}^{2}\rangle }_{1}},\,2\sqrt{{\langle {z}^{2}\rangle }_{1}})$ contains more than the 75% of the total power. Equivalently, outside Ω (i.e. ${\mathbb{R}}-{\rm{\Omega }}$) the following inequality holds

21 $$\frac{{\int }_{{\mathbb{R}}-{\rm{\Omega }}}\,|{{\bf{E}}}_{1}\mathrm{(0},z{)|}^{2}\,{\rm{d}}z}{{\int }_{-\infty }^{\infty }\,|{{\bf{E}}}_{1}\mathrm{(0},z{)|}^{2}\,{\rm{d}}z}\le \frac{{\langle {z}^{2}\rangle }_{1}}{{L}^{2}}.$$

Regarding **E**_2_(0, *z*), combining Eqs (18) and (19b) we get

22 $$|{{\bf{E}}}_{2}\mathrm{(0,}\,z{)|}^{2}=\frac{|\tilde{{\bf{F}}}({\alpha }_{0}{)|}^{2}}{4}{\mathrm{(1}-{\alpha }_{0})}^{2}\,[{{\rm{sinc}}}^{{\rm{2}}}\,(\frac{kz}{2}\mathrm{(1}-{\alpha }_{0}))+{j}_{1}^{2}\,(\frac{kz}{2}\mathrm{(1}-{\alpha }_{0}))].$$

where *j*_1_ is the spherical Bessel function of first order. The second-order intensity moment for the function **E**_2_(0, *z*) cannot be calculated because of the divergence of the integral. However, its power content can be bounded using the asymptotic behaviour for *j*_1_: in fact, it can be proven that^30^.

23 $${\int }_{{\mathbb{R}}-{\rm{\Omega }}}\,|{{\bf{E}}}_{2}\mathrm{(0,}\,z{)|}^{2}\,{\rm{d}}z\le \frac{\mathrm{4|}\tilde{{\bf{F}}}({\alpha }_{0}{)|}^{2}}{L\,{k}^{2}}.$$

Using the content-ratio formula introduced in Eq. (21), the following condition is obtained

24 $$\begin{array}{rcl}1-q & = & \frac{{\int }_{{\mathbb{R}}-{\rm{\Omega }}}\,|{\bf{E}}\mathrm{(0},\,z{)|}^{2}\,{\rm{d}}z}{{\int }_{-\infty }^{\infty }\,|{\bf{E}}\mathrm{(0,}\,z{)|}^{2}\,{\rm{d}}z}\\  & \le  & 2\frac{{\int }_{{\mathbb{R}}-{\rm{\Omega }}}\,|{{\bf{E}}}_{1}\mathrm{(0,}\,z{)|}^{2}\,{\rm{d}}z}{{\int }_{-\infty }^{\,\infty }\,|{\bf{E}}\mathrm{(0,}\,z{)|}^{2}\,{\rm{d}}z}+2\frac{{\int }_{{\mathbb{R}}-{\rm{\Omega }}}\,|{{\bf{E}}}_{2}\mathrm{(0,}\,z{)|}^{2}\,{\rm{d}}z}{{\int }_{-\infty }^{\infty }\,|{\bf{E}}\mathrm{(0,}\,z{)|}^{2}\,{\rm{d}}z}.\end{array}$$

Note that when either **E**_1_ or **E**_2_ is equals to zero, prefactors 2 can be omitted obtaining a more accurate relationship between the 1 − *q* value and the on-axis power-content. Combining the previous inequality with Eqs (21) and (23), the following condition holds:

25 $$1-q\le \frac{2}{I{L}^{2}{k}^{2}}[{\int }_{{\alpha }_{0}}^{1}\,{|\frac{{\rm{d}}\tilde{{\bf{F}}}(\alpha )}{{\rm{d}}\alpha }|}^{2}\,{\rm{d}}\alpha +\frac{|\tilde{{\bf{F}}}({\alpha }_{0}{)|}^{2}}{{\mathrm{(1}-{\alpha }_{0})}^{2}}]+\frac{\mathrm{8|}\tilde{{\bf{F}}}({\alpha }_{0}{)|}^{2}}{IL{k}^{2}}.$$

For the particular case of fields that vanish at the entrance pupil boundary (i.e. $\tilde{{\bf{F}}}({\alpha }_{0})=0$ and therefore **E**_2_(0, *z*) = 0), the value of *L* that guarantees a power content of at least 75% of the total power within the region Ω is given by

26 $$L=\frac{\lambda }{\pi }{[\frac{{\int }_{{\alpha }_{0}}^{1}{|\frac{{\rm{d}}\tilde{{\bf{F}}}(\alpha )}{{\rm{d}}\alpha }|}^{2}{\rm{d}}\alpha }{{\int }_{{\alpha }_{0}}^{1}|\tilde{{\bf{F}}}(\alpha {)|}^{2}{\rm{d}}\alpha }]}^{1/2}.$$

### Measurement of the modulation response of the displays

**Figure 7:**
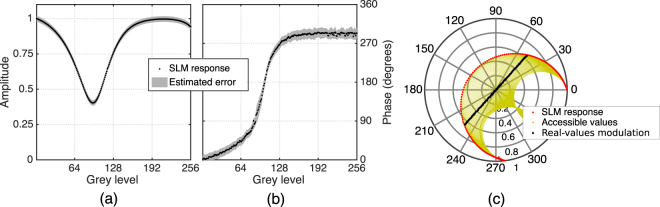
SLM modulation response: (**a**) amplitude, (**b**) phase, (**c**) holographically encoded accessible values.

Figure 7(a,b) show the modulated amplitude and phase values as a function of the displayed grey-level; the grey bands show the error associated to each curve. Figure 7(c) illustrates the modulation complex plane. Red dots display the information contained in 7(a,b) in polar form. It is apparent that only a few values can be modulated using a single display. However, holographic codification methods such as the cell-based double-pixel hologram^22^ enables to generate full-complex modulation. Yellow dots show accessible values using this holographic procedure. Then, a subset of this points laying on a straight line are selected (black dots); they are used to generate the holograms displayed on Fig. 4.
